# Soil classification in the Sudan Savanna using sentinel products and topographic information with machine learning models

**DOI:** 10.1038/s41598-026-46259-6

**Published:** 2026-03-27

**Authors:** Win Sithu Maung, Ikazaki Kenta, Sakai Toru, Simporé Saïdou, Zerbo Lamine, Kone Nicolas

**Affiliations:** 1 Japan International Research Center for Agricultural Sciences (JIRCAS), Tsukuba, 305-8686 Ibaraki Japan; 2 Institut de l’Environnement et de Recherches Agricoles (INERA), Ouagadougou, Burkina Faso

**Keywords:** Soil classification, Machine learning, Sentinel image, Topography feature, Sudan Savanna, Ecology, Ecology, Environmental sciences

## Abstract

Accurate soil information is crucial for sustainable agricultural planning and land management, particularly in data-scarce regions, such as the Sudan Savanna, the largest sorghum-producing area in Africa. A recent study reported that soils in this region corresponded well with the topography, having formed primarily through erosion–deposition processes, resulting in systematic variation in soil types along the landscape. Therefore, this study compared the performances of three machine learning models, i.e., Random Forest (RF), Extreme Gradient Boosting (XGBoost), and Support Vector Machine (SVM), for soil classification based on multisource remote sensing and topographic data. Ground-truth data with four different soil types, Lixisols, Petric Plinthosols, Pisoplinthic Petric Plinthosols, and Gleysols, were used to train and validate the models using 19 remote sensing-derived covariates including Sentinel-1 SAR, Sentinel-2 bands, spectral indices, and Topographic Wetness Index. Machine learning classification was analyzed under different scenarios of remote sensing feature combination. Results showed that the XGBoost with the selected feature combination achieved the highest performance with an overall accuracy of 78.9%, followed by RF (72.3%) and SVM (65.2%). Among the selected features, topographic parameters appeared the most important and provided complementary information for accurate soil classification. This study demonstrates the effectiveness of integrating optical, radar, and topographic information for soil mapping and provides a valuable management tool to support agricultural and environmental strategies in the Sudan Savanna.

## Introduction

 Soil is a fundamental resource for agriculture, ecosystems, and human well-being. Soil classification provides a basis for sustainable land-use planning, crop management, and the assessment of land degradation^1^. Therefore, the development of soil maps is particularly urgent in sub-Saharan African (SSA) countries that suffer from food shortages and soil degradation. However, this development has been delayed, resulting in a lack of fundamental knowledge for policy and land management decisions^2^. For example, in Burkina Faso, which includes the Sudan Savanna, the largest sorghum-producing region in Sub-Saharan Africa, local farmers had access to 1:100,000 scale soil maps updated by BUNASOLS between the mid-1990s and the mid-2000s (https://www.bunasols.bf/index.php/2017-09-13-14-27-15/2017-09-10-16-02-03/2017-09-10-16-03-01 (accessed December 16, 2025). However, as BUNASOLS itself states, these maps are too large to be useful for farmers who plan land use and other activities. Therefore, farmers rely on local soil knowledge for field management decision-making^3,4^. However, local soil knowledge is typically qualitative, site specific, and not spatially continuous, making it difficult to standardize, scale up, integrate into regional planning and digital decision-support systems. Consequently, Ikazaki et al.^5,6^ have reported yield-limiting factors and proposed optimal management practices for sorghum cultivation for each dominant soil type in the Sudan Savanna based on international taxonomy; however, local farmers cannot apply these management practices according to the soil type in their own fields. This delay in soil mapping was due to the lack of suitable covariates and the absence of updated high-quality field measurements required for model calibration and validation^7,8^. However, training soil specialists and establishing a sustainable soil monitoring system in each country requires considerable time and expense. Therefore, developing alternative methods to create high-resolution soil maps on a regional scale is an urgent priority^8,9^.

In the Sudan Savanna, soil types have been reported to correspond well with topography. This is because the soil-forming factors are similar across large areas in this region^10^. The parent materials consist mainly of acidic rocks such as granitoids and their metamorphic products^11^. The topography features gently rolling hills with elevations of 250–350 m and slopes of 1% (i.e., peneplain). The climate has alternated between humid and arid periods^12^, but is currently tropical and semi-arid. Vegetation has changed over time, alternating between humid and arid periods, resulting in forests, savannas, or their combination^13^. The soil formation time is exceptionally long because of the stable topography of the region. Therefore, it may be possible to predict soil types with high accuracy using topographic information in the Sudan Savanna, which is critical for food security in the SSA region.

Additionally, recent advances in Earth observations have provided opportunities to improve soil-type mapping in data-scarce environments^14–17^. Sentinel satellites offer free and frequent coverage using different optical and radar sensors. Sentinel-2 delivers optical data across multiple spectral bands. Sentinel-1 provides radar backscatter information that is sensitive to surface roughness and soil moisture, with the advantage of being unaffected by cloud cover. Many studies have explored land cover and vegetation status using Sentinel-1 and 2 data in the Savanna region^18–23^. In addition to satellite imagery, topographic information derived from digital elevation models has proven valuable for soil properties and landscape mapping^24,25^. Terrain attributes, such as the Topographic Wetness Index (TWI), which represents potential water accumulation, are strongly related to soil moisture regimes, drainage conditions, and pedogenesis^26–28^.

Along with these developments in remote sensing data, machine and deep learning techniques have transformed digital soil mapping by offering flexible and powerful classification approaches^9,15,29–35^. Unlike traditional statistical models, machine learning algorithms can capture nonlinear relationships and complex interactions among predictors. These models have been successfully applied to soil property predictions and land-cover classifications in many regions^30,35–39^. To date, no study has systematically evaluated soil classification using machine learning by jointly integrating multisource remote sensing data-optical, radar, and topographic information-in the Sudan Savanna. This represents a critical knowledge gap for data-scarce semi-arid regions where field surveys are limited but satellite observations are abundant.

Building on the urgent necessity of a soil map for the Sudan savanna, this study aimed to develop a soil classification method using the potential applications of remote sensing and machine learning in the Central Plateau of Burkina Faso. We specifically evaluated three widely used machine learning algorithms such as Random Forest (RF), Extreme Gradient Boosting (XGBoost), and Support Vector Machine (SVM) because they are well suited for classification problems with limited and heterogeneous training data and have demonstrated strong performance in environmental mapping studies. By comparing three machine learning models using multisource predictors derived from Sentinel-1, Sentinel-2, and spectral and topographic indices, this study provides insights into the potential of remote sensing-based soil classification in data-scarce regions and highlights practical implications for agricultural management and sustainable land use in the Sudan Savanna. The proposed scenario is expected to be a scalable approach for other parts of the Sudan Savanna and beyond. From a practical perspective, this study provides updated soil type information that can support agricultural planning, soil management, and environmental monitoring.

## Methods

### Study area and field survey

**Fig. 1:**
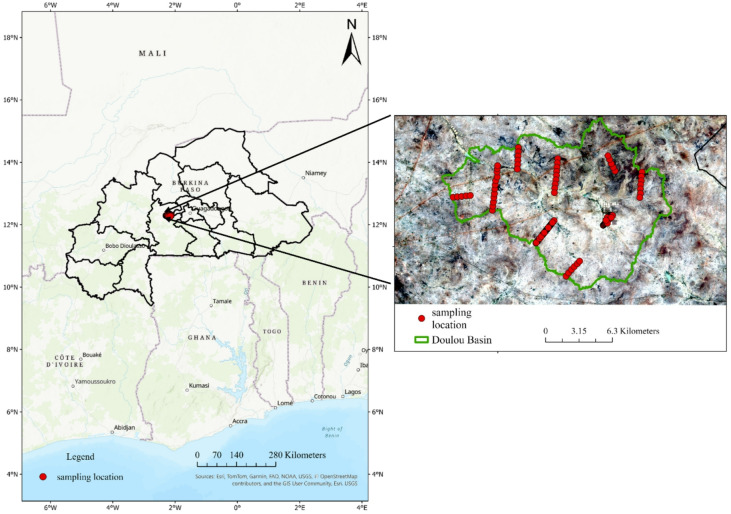
Study site and soil sampling locations in Central Burkina Faso. The map was created using USGS OpenStreetMap and Sentinel-2 imagery (2020), and processed in ArcGIS Pro (version 3.6.0; https://www.esri.com/arcgis).

The study area was the Doulou basin where the Saria station of the Institute of Environment and Agricultural Research is located (N 12°16ʹ, W 2°09ʹ; 300 m above sea level) (Fig. 1)^40^. The Doulou basin is located on the Central Plateau of Burkina Faso, a typical region of the Sudan Savanna. The rainy period mostly occurs between June and September, with a mean annual rainfall of 800 mm. The mean annual temperature is 28 °C, and mean annual potential evaporation is between 1,700 and 2,000 mm. The major land use type is farmed parkland, where crops are grown alongside the conservation of native or useful trees. A recent study Takenaka et al.^40^ recorded 26 woody vegetation types in the study area and revealed that tree species were more abundant on Lixisols at lower topography. *Vittelaria paradoxa* and *Lannea microcarpa* were the dominant species across all soil types.

**Fig. 2:**
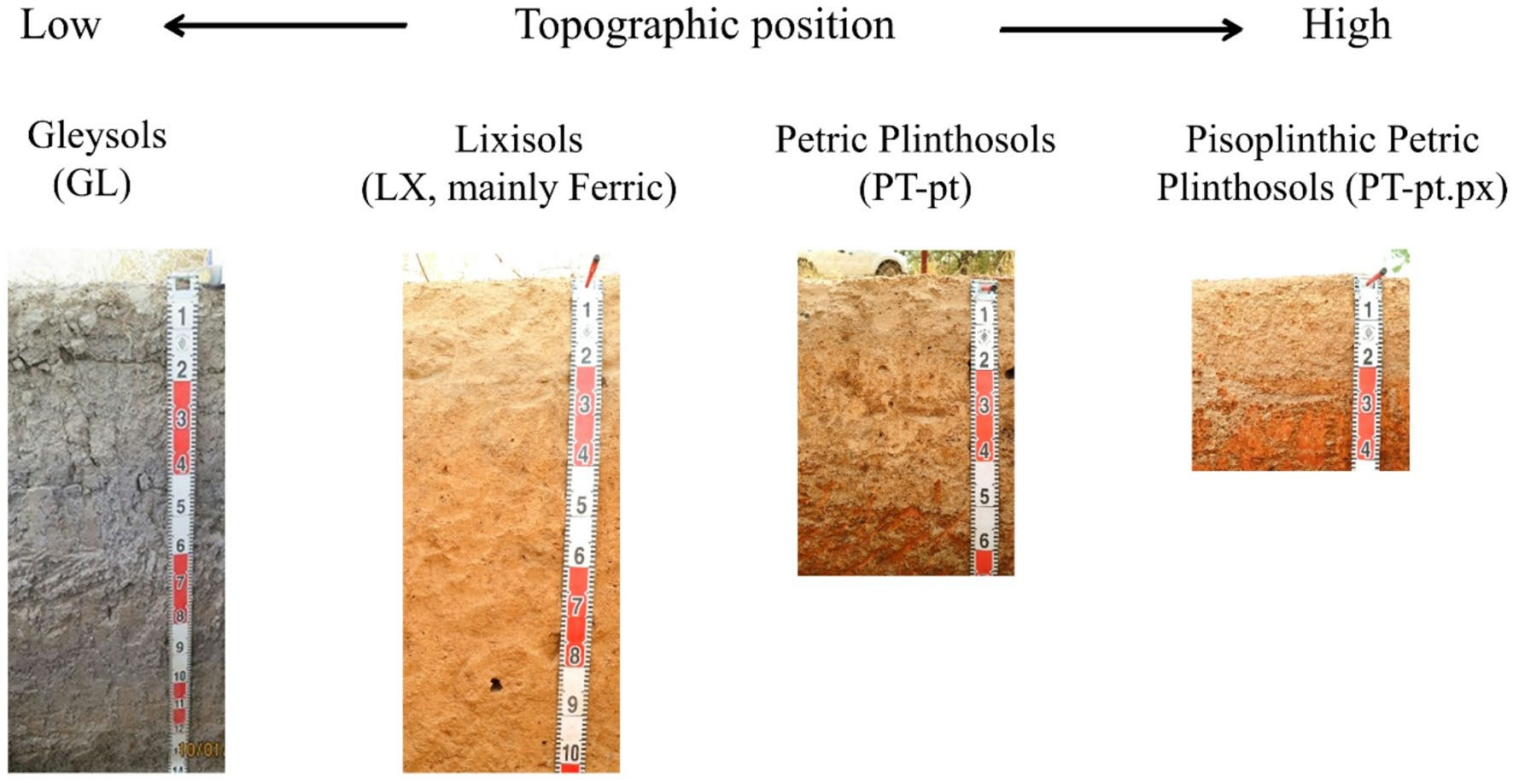
Representative soil profiles and corresponding topographic positions of the four ground-truth soil types: LX, PT-pt, PT-pt.px, and GL illustrating their distinct color, texture, and landscape settings.

Soil-profile surveys were conducted from 2016 to 2018 along nine line transects ranging from 1.2 to 4.2 km in length, established within the watershed from the riverbed toward the plateau (Fig. 2). Soil profiles were identified at 141 locations at intervals of 50–400 m (61 with soil pits and 80 using a hand auger). Soil samples taken from each horizon were then analyzed as previously described^41^. The soils were classified according to the WRB soil classification system (IUSS Working Group, 2015). Four main soil types were identified: Lixisols (LX; mainly ferric), Petric Plinthosols (PT-pt), Pisoplinthic Petric Plinthosols (PT-pt.px), and Gleysols (GL) (Table 1; Fig. 2). The ground-truth data from the four different soil types were utilized for training and testing the machine learning models.

**Table 1:** Description of the four major soil types identified in the study area.

Soil Type	Code	No. of Samples	Key Characteristics
Lixisols	LX	38	Mainly Ferric, sandy topsoil, deep effective soil depth (~ 100 cm), relatively fertile, found primarily in the lower or toe slopes
Petric Plinthosols	PT-pt	47	Sandy topsoil, moderate effective soil depth (~ 50 cm), found primarily in the middle slope
Pisoplinthic Petric Plinthosols	PT-pt.px	48	Gravelly topsoil, shallow effective soil depth (~ 25 cm), relatively unfertile, found primarily in the upper slope or on the plateau
Gleysols	GL	8	Poorly drained soils with gleyic features (reduced conditions), found only in the riverbed

For soil physical and chemical properties of LX, PT-pt, and PT-pt.px, please refer to Ikazaki et al.^5^.

### Remote sensing data

For the remote sensing inputs, Sentinel-1 SAR backscatter coefficients (σVV and σVH) and their derived polarization combinations (σVH–σVV, σVH + σVV, and σVH/σVV) were used together with Sentinel-2 multispectral bands obtained from the European Space Agency archives^42,43^. Using the related spectral bands of Sentinel-2, Normalized Difference Vegetation Index, Normalized Difference Water Index, Modified Normalized Difference Water Index, and Normalized Difference Soil Index were calculated to enhance vegetation and moisture-related soil characteristics. The TWI was derived from Advanced Land Observing Satellite (ALOS) World 3D (AW3D) high-resolution global DEM data with 5 m resolution. The AW3D DEM offers a detailed and high-resolution three-dimensional representation of the Earth’s surface. It is generated from PRISM sensor data acquired by the ALOS operated by the Japan Aerospace Exploration Agency. All the spatial information was integrated and matched with field-collected soil classes to build the final dataset for analysis. All features used in this study are listed in Table 2.

**Table 2:** Remote sensing features used in soil classification in this study.

Product	Feature	Wavelength information/Formula	Reference
Sentinel-1	VH	Horizontally polarized backscatter	^43^
	VV	Vertically polarized backscatter	
	VV-VH	SAR transformation	^44^
	VV + VH		
	VV/VH		
Sentinel-2	B2-Blue	490 nm	^42^
	B3-Green	560 nm	
	B4-Red	655 nm	
	B5-Red Edge1	705 nm	
	B6-Red Edge2	740 nm	
	B7-Red Edge3	783 nm	
	B8- NIR	842 nm	
	B11-Short Wave Infra-Red) (SWIR-1)	1610 nm	
	B12-Short Wave Infra-Red) (SWIR-2)	2190 nm	
	Normalized Difference Vegetation Index (NDVI)	$\:\frac{\mathrm{B}8-\mathrm{B}4}{\mathrm{B}8+\mathrm{B}4}$	^45^
	Normalized Difference Water Index (NDWI)	$\:\frac{\mathrm{B}3-\mathrm{B}8}{\mathrm{B}3+\mathrm{B}8}$	^46^
	Modified Normalized Difference Water Index (MNDWI)	$\:\frac{\mathrm{B}3-\mathrm{B}11}{\mathrm{B}3+\mathrm{B}11}$	^47^
	Normalized Difference Soil Index (NDSI)	$\:\frac{\mathrm{B}11-\mathrm{B}8}{\mathrm{B}11+\mathrm{B}8}$	^48^
AW3D DEM	Topographic Wetness Index (TWI)	ln(α/tanβ)	^49^

### Machine learning classification

Machine learning techniques were applied to classify soil types using combined features derived from multisource remote sensing data and topographic information. Three supervised classification algorithms, Random Forest (RF), Extreme Gradient Boosting (XGBoost), and Support Vector Machine (SVM), were implemented and compared in this study. First, we set various scenarios of remote sensing derived feature combinations to determine the optimal approach for soil classification. The machine learning experiments were implemented in Python 3.12 using the scikit-learn and XGBoost libraries. All models were trained using default hyperparameters described in Table 3 and validated using ground-truth soil data, corresponding feature values extracted from the Sentinel products, and a digital elevation-derived index.

#### RF

Breiman^50^developed the RF algorithm, which is an ensemble learning technique that combines the predictions of multiple decision trees. Each tree is built from a random subset of training samples and predictor variables, which introduces diversity and prevents overfitting. The final classification is obtained through majority voting among all trees. RF performs well with nonlinear and high-dimensional data, and is widely used in remote sensing because of its robustness and ability to assess the relative importance of input features. In addition, RF is relatively robust to class imbalance because each tree is trained using bootstrap samples and random feature selection, which helps reduce bias toward majority classes. Grimm et al^39^. explored digital soil mapping using an RF model for soil organic carbon prediction in Barro Colorado Island. Similarly, Wiesmeier et al^51^. proposed an RF algorithm combined with Classification and Regression Trees (CART) as a promising approach for modeling soil properties in semi-arid ecosystems.

#### XGBoost

The XGBoost model is an advanced implementation of gradient boosting that sequentially constructs decision trees, with each new tree trained to correct the errors of the previous ensemble. This minimizes a regularized objective function that controls both model accuracy and complexity, thereby improving generalization. XGBoost is computationally efficient and capable of modeling complex nonlinear interactions between spectral and topographic variables. In this study, XGBoost achieved the highest classification performance, highlighting its suitability for integrating multisource remote sensing data into soil mapping. Similar to RF, XGBoost is less sensitive to moderate class imbalance due to its ensemble structure and regularization mechanism, which help stabilize learning even when class frequencies differ. This makes it suitable for soil classification problems with uneven sample distribution among classes. Khalaf et al^52^. compared the RF and XGBoost models for digital soil mapping of soil organic matter in Northern Iraq, and reported that XGBoost produced higher prediction accuracy than the RF model. The estimation of soil loss due to water erosion was also improved in a previous study^53^ using a hybrid CNN-XGBoost approach.

#### SVM

The SVM algorithm is a supervised algorithm that identifies an optimal hyperplane by separating the data points of different classes in a multidimensional feature space. By applying kernel functions, such as the radial basis function (RBF) kernel, SVM can handle nonlinear relationships between predictor variables and class labels. SVM is effective for small- to medium-sized datasets and offers strong generalization capabilities. In this study, StandardScaler feature scaling was applied prior to training the SVM model to normalize the predictor variables, as SVM performance is sensitive to differences in feature magnitude. In addition, the RBF kernel was used to enhance the separability of soil classes and improve classification reliability in heterogeneous savanna landscapes. Kovačević et al^54^. proposed an SVM model with Leave-One-Out Cross-Validation (LOOCV) for soil type classification and soil property estimation in eastern Serbia. Pereira et al^55^. developed an SVM model to predict soil attributes and demonstrated its superiority over other machine learning models. In contrast, SVM can be more sensitive to class imbalance because the optimal separating hyperplane may be influenced disproportionately by majority classes. Therefore, SVM performance was carefully evaluated using class-wise accuracy metrics in addition to overall accuracy.

After reviewing the potential applications of various machine learning models in soil-related analysis, we compared the three models for soil classification. To ensure an unbiased evaluation of model performance, our study also employed the LOOCV approach for soil classification. In LOOCV, each individual sample was used once as validation data, while the remaining samples were used for training. This process was repeated until each sample served as a validation point, and the final accuracy was computed as the average across all iterations. Although computationally intensive, LOOCV provides a robust estimate of model performance for relatively small datasets, thereby reducing bias and overfitting. This strategy also allowed each minority-class sample, particularly GL class, to contribute to both training and validation phases, which is beneficial when working with imbalanced soil datasets.

**Table 3:** Hyperparameters used in machine learning models in this study.

Model	Hyperparameter	Value	Description
Random Forest	n_estimators	200	Number of trees
	max_depth	None	Nodes expanded until pure
	min_samples_split	2	Minimum samples to split
	min_samples_leaf	1	Minimum samples per leaf
	random_state	42	Reproducibility seed
XGBoost	objective	multi: softmax	Multiclass classification
	num_class	4	Number of soil classes
	eval_metric	mlogloss	Multiclass log loss
	learning_rate	0.3 (default)	Step size shrinkage
	max_depth	6 (default)	Tree depth
	n_estimators	100 (default)	Boosting rounds
	random_state	42	Reproducibility seed
SVM	kernel	RBF	Nonlinear kernel
	C	1.0	Regularization parameter
	gamma	scale	Kernel coefficient
	random_state	42	Seed

### Model evaluation

The model performance was quantitatively assessed using standard classification accuracy metrics derived from the confusion matrix. The overall accuracy (OA) was computed as the ratio of correctly classified samples to the total number of samples, providing an intuitive measure of the model’s predictive capability ($\:\mathrm{E}\mathrm{q}\mathrm{u}\mathrm{a}\mathrm{t}\mathrm{i}\mathrm{o}\mathrm{n}\:1$)^56^. Additionally, the confusion matrix enabled a detailed evaluation of class-specific performance by identifying omission and commission errors among different soil types.

Further evaluation was conducted using additional performance metrics, including F1-score ($\:\mathrm{E}\mathrm{q}\mathrm{u}\mathrm{a}\mathrm{t}\mathrm{i}\mathrm{o}\mathrm{n}\:2$), precision ($\:\mathrm{E}\mathrm{q}\mathrm{u}\mathrm{a}\mathrm{t}\mathrm{i}\mathrm{o}\mathrm{n}\:3$), and recall $\:(\mathrm{E}\mathrm{q}\mathrm{u}\mathrm{a}\mathrm{t}\mathrm{i}\mathrm{o}\mathrm{n}\:\:4$), to capture the accuracy and reliability of the proposed classification model^57^. Finally, feature importance analysis was performed to determine the most influential variables contributing to soil type discrimination. This analysis provided valuable insights into the relative contributions of spectral, radar, and terrain-derived attributes in predicting soil distribution patterns across the Sudan Savanna region.

1 $$\:\mathrm{O}\mathrm{A}\:=\:\frac{\mathrm{N}\mathrm{u}\mathrm{m}\mathrm{b}\mathrm{e}\mathrm{r}\:\mathrm{o}\mathrm{f}\:\mathrm{c}\mathrm{o}\mathrm{r}\mathrm{r}\mathrm{e}\mathrm{c}\mathrm{t}\:\mathrm{p}\mathrm{r}\mathrm{e}\mathrm{d}\mathrm{i}\mathrm{c}\mathrm{t}\mathrm{i}\mathrm{o}\mathrm{n}}{\mathrm{T}\mathrm{o}\mathrm{t}\mathrm{a}\mathrm{l}\:\mathrm{n}\mathrm{u}\mathrm{m}\mathrm{b}\mathrm{e}\mathrm{r}\:\mathrm{o}\mathrm{f}\:\mathrm{p}\mathrm{r}\mathrm{e}\mathrm{d}\mathrm{i}\mathrm{c}\mathrm{t}\mathrm{i}\mathrm{o}\mathrm{n}}$$

2 $$\:\mathrm{F}1\:=\:2\times\:\frac{\mathrm{P}\mathrm{r}\mathrm{e}\mathrm{c}\mathrm{i}\mathrm{s}\mathrm{i}\mathrm{o}\mathrm{n}\times\:\mathrm{R}\mathrm{e}\mathrm{c}\mathrm{a}\mathrm{l}\mathrm{l}}{\mathrm{P}\mathrm{r}\mathrm{e}\mathrm{c}\mathrm{i}\mathrm{s}\mathrm{i}\mathrm{o}\mathrm{n}+\mathrm{R}\mathrm{e}\mathrm{c}\mathrm{a}\mathrm{l}\mathrm{l}}$$

3 $$\:\mathrm{P}\mathrm{r}\mathrm{e}\mathrm{c}\mathrm{i}\mathrm{s}\mathrm{i}\mathrm{o}\mathrm{n}\:=\:\frac{\mathrm{T}\mathrm{r}\mathrm{u}\mathrm{e}\:\mathrm{p}\mathrm{o}\mathrm{s}\mathrm{i}\mathrm{t}\mathrm{i}\mathrm{v}\mathrm{e}}{\mathrm{T}\mathrm{r}\mathrm{u}\mathrm{e}\:\mathrm{p}\mathrm{o}\mathrm{s}\mathrm{i}\mathrm{t}\mathrm{i}\mathrm{v}\mathrm{e}+\mathrm{F}\mathrm{a}\mathrm{l}\mathrm{s}\mathrm{e}\:\mathrm{p}\mathrm{o}\mathrm{s}\mathrm{i}\mathrm{t}\mathrm{i}\mathrm{v}\mathrm{e}}$$

4 $$\:\mathrm{R}\mathrm{e}\mathrm{c}\mathrm{a}\mathrm{l}\mathrm{l}=\:\frac{\mathrm{T}\mathrm{r}\mathrm{u}\mathrm{e}\:\mathrm{p}\mathrm{o}\mathrm{s}\mathrm{i}\mathrm{t}\mathrm{i}\mathrm{v}\mathrm{e}}{\mathrm{T}\mathrm{r}\mathrm{u}\mathrm{e}\:\mathrm{p}\mathrm{o}\mathrm{s}\mathrm{i}\mathrm{t}\mathrm{i}\mathrm{v}\mathrm{e}+\mathrm{F}\mathrm{a}\mathrm{l}\mathrm{s}\mathrm{e}\:\mathrm{n}\mathrm{e}\mathrm{g}\mathrm{a}\mathrm{t}\mathrm{i}\mathrm{v}\mathrm{e}}$$

**Fig. 3:**
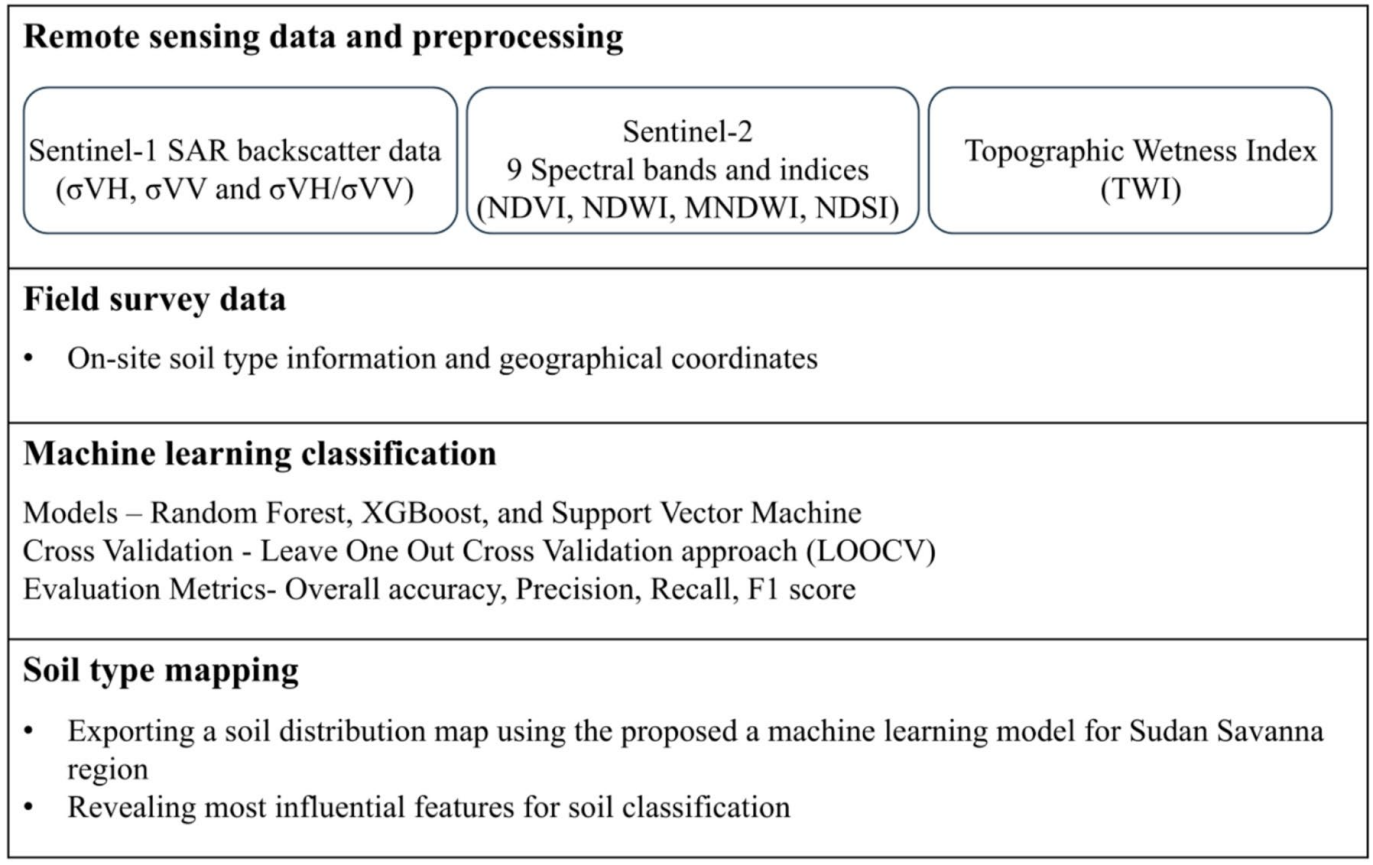
Workflow of soil classification using machine learning models and multisource remote sensing dataset.

The overall workflow of this study is shown in Fig. 3. Remote sensing data from multiple sources were collected and preprocessed to derive spectral and topographic features relevant to soil properties. Using ground-truth data containing soil type information and the corresponding GPS coordinates, machine learning models were trained to classify soil types. After identifying the best-performing model, the proposed approach was applied to generate a spatially continuous soil type map for the entire study area within the Sudan Savanna region.

## Results

### Soil classification using different scenarios

**Fig. 4:**
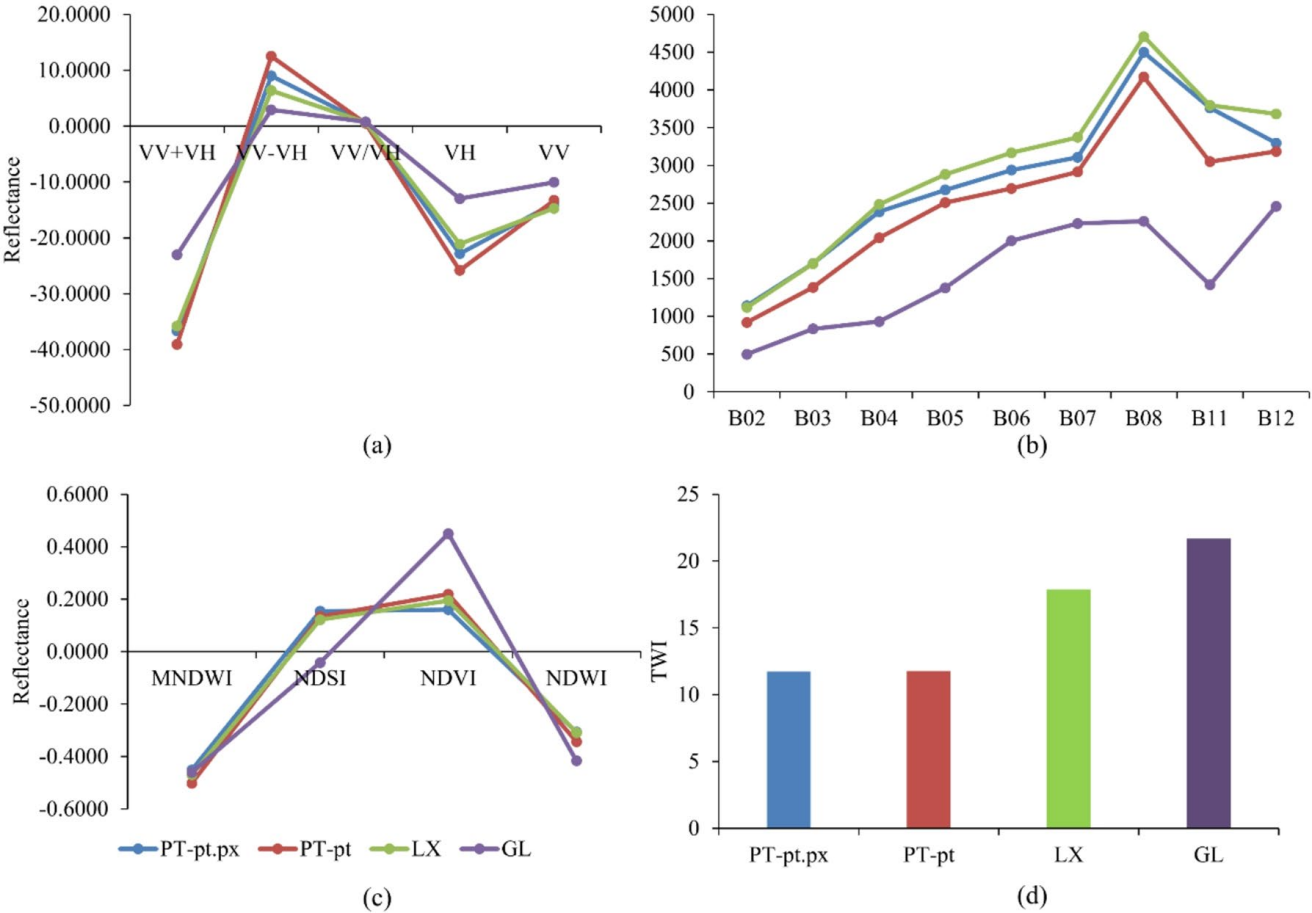
Comparison of mean reflectance, spectral indices, and TWI among the four major soil types in the Sudan Savanna: Lixisols (LX), Petric Plinthosols (PT-pt), Pisoplinthic Petric Plinthosols (PT-pt.px), and Gleysols (GL).

Before conducting the classification analysis, the reflectance characteristics of the Sentinel-1 and Sentinel-2 data, as well as the derived spectral and topographic indices, were examined to assess their relationships with the four major soil types: LX, PT-pt, PT-pt.px, and GL (Fig. 4). Distinct spectral and backscatter patterns were observed across the soil types, particularly in the short-wave infrared (SWIR) and red-edge bands, which are sensitive to soil mineral composition, texture, and moisture content. Similarly, the TWI exhibited clear separability among soil classes, reflecting differences in hydrological and geomorphological conditions that influence soil formation and properties. These findings indicated that both spectral and terrain-related variables provide complementary information relevant to soil discrimination.

The classification performance of the three machine learning models (RF, XGBoost, and SVM) was evaluated using various combinations of multisource remote sensing and topographic data, as listed in Table 4. Among the scenarios, Scenario 5 (Sentinel-2 + TWI) produced the highest overall accuracy. In this scenario, the XGBoost model achieved the best performance, with an accuracy of 78.7%, followed by RF (72.3%), whereas the SVM showed slightly lower (65.2%) accuracy. The strong performance of XGBoost highlights its ability to capture nonlinear relationships between spectral variables and topographic moisture patterns represented by the TWI.

The integration of Sentinel-2 spectral bands with the TWI consistently improved classification accuracy compared to scenarios using Sentinel-1 alone (Scenarios 1 and 4) or Sentinel-1 combined with Sentinel-2 (Scenarios 3 and 6). Notably, all scenarios incorporating the TWI (Scenarios 4–6) outperformed those without, confirming the added value of information regarding water flow for soil discrimination. In contrast, models relying only on Sentinel-1 SAR data (Scenario 1) showed the lowest accuracy, reflecting the limited sensitivity of C-band SAR to key soil properties relative to optical and topographic predictors.

Based on a detailed classification report for the best-performing XGBoost model under Scenario 5 (Table 4), the PT-pt.px class exhibited a strong predictive performance, with a precision of 0.83, recall of 0.78, and an F1-score of 0.80. The PT-pt class showed a similarly high performance (precision: 0.78; recall: 0.84; and F1-score: 0.81). For the LX and GL classes, the precision, recall, and F1-scores were 0.73, 0.73, and 0.73 and 0.86, 0.75, and 0.79, respectively. The macro-averaged metrics across all classes had a precision of 0.80, recall of 0.78, and an F1-score of 0.79, indicating that the XGBoost model effectively captured the spatial variability of soil types in the heterogeneous savanna landscape (Table 5).

**Table 4:** Classification results produced by machine learning models based on different feature combinations.

Scenarios	Feature combination	Classification accuracy (%)		
		RF	XGBoost	SVM
1	Sentinel-1	44.7	42.6	51.7
2	Sentinel-2	61.7	54.6	52.4
3	Sentinel-1 + Sentinel-2	56.7	53.9	53.2
4	Sentinel-1 + TWI	60.3	61.7	63.1
5	Sentinel-2 + TWI	72.3	**78.7**	65.2
6	Sentinel-1 + Sentinel-2 + TWI	70.9	73.8	61.7

**Table 5:** Classification performance metrics (precision, recall, and F1-score) for the four soil types using the XGBoost model (Scenario 5).

Class	Soil Type	Precision	Recall	F1-Score	Support
1	PT-pt.px (Pisoplinthic Petric Plinthosols)	0.83	0.78	0.80	45
2	PT-pt (Petric Plinthosols)	0.78	0.84	0.81	51
3	LX (Lixisols)	0.73	0.73	0.73	37
4	GL (Gleysols)	0.86	0.75	0.80	8
**Total**					**141**
**Overall Accuracy**					**0.79**
**Average**		**0.80**	**0.78**	**0.79**	

### Feature importance analysis

**Fig. 5:**
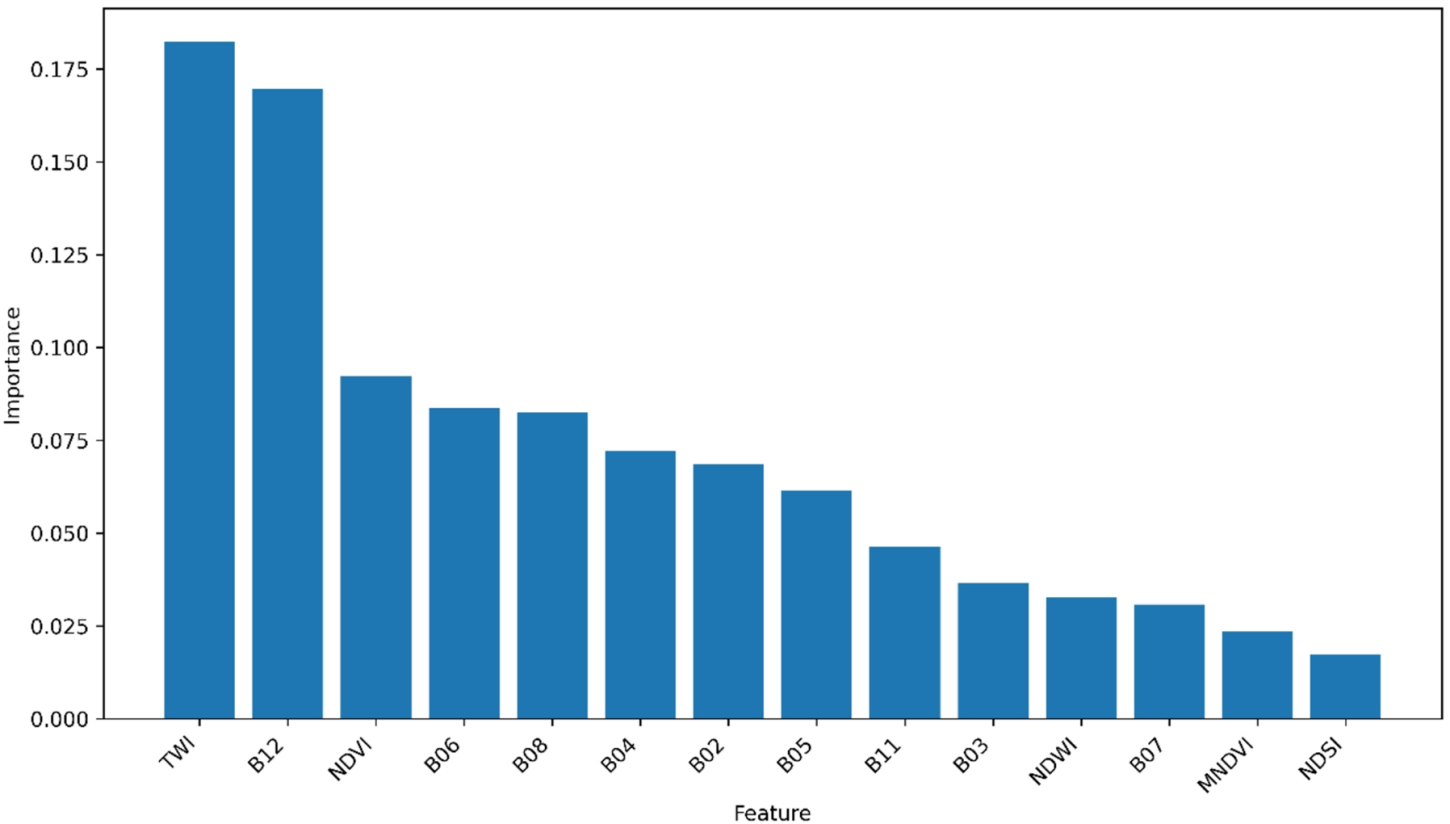
Feature importance derived from the XGBoost model for soil type classification in the Sudan Savanna.

To identify the most influential predictors for soil classification, we analyzed the feature importance derived from the best-performing XGBoost model under Scenario 5. Feature importance was calculated using the gain metric (default setting in XGBoost), which represents the average reduction in the loss function contributed by splits involving each feature. The analysis revealed that topographic and spectral variables contributed differentially to model performance, highlighting the complementary role of terrain and optical data in soil mapping (Fig. 5).

Among all the features, the TWI was the most important predictor for soil classification. The TWI captures spatial variations in soil water movement, which strongly influences soil development, organic matter content, and erosion processes. Following the TWI, the Sentinel-2 SWIR band 12 was the next most influential feature. The SWIR band is sensitive to soil mineralogy, moisture content, and texture, facilitating the discrimination of soil types based on their spectral characteristics. Other spectral bands and indices provided additional information related to vegetation–soil interactions and surface moisture conditions.

### Mapping soil type distribution

**Fig. 6:**
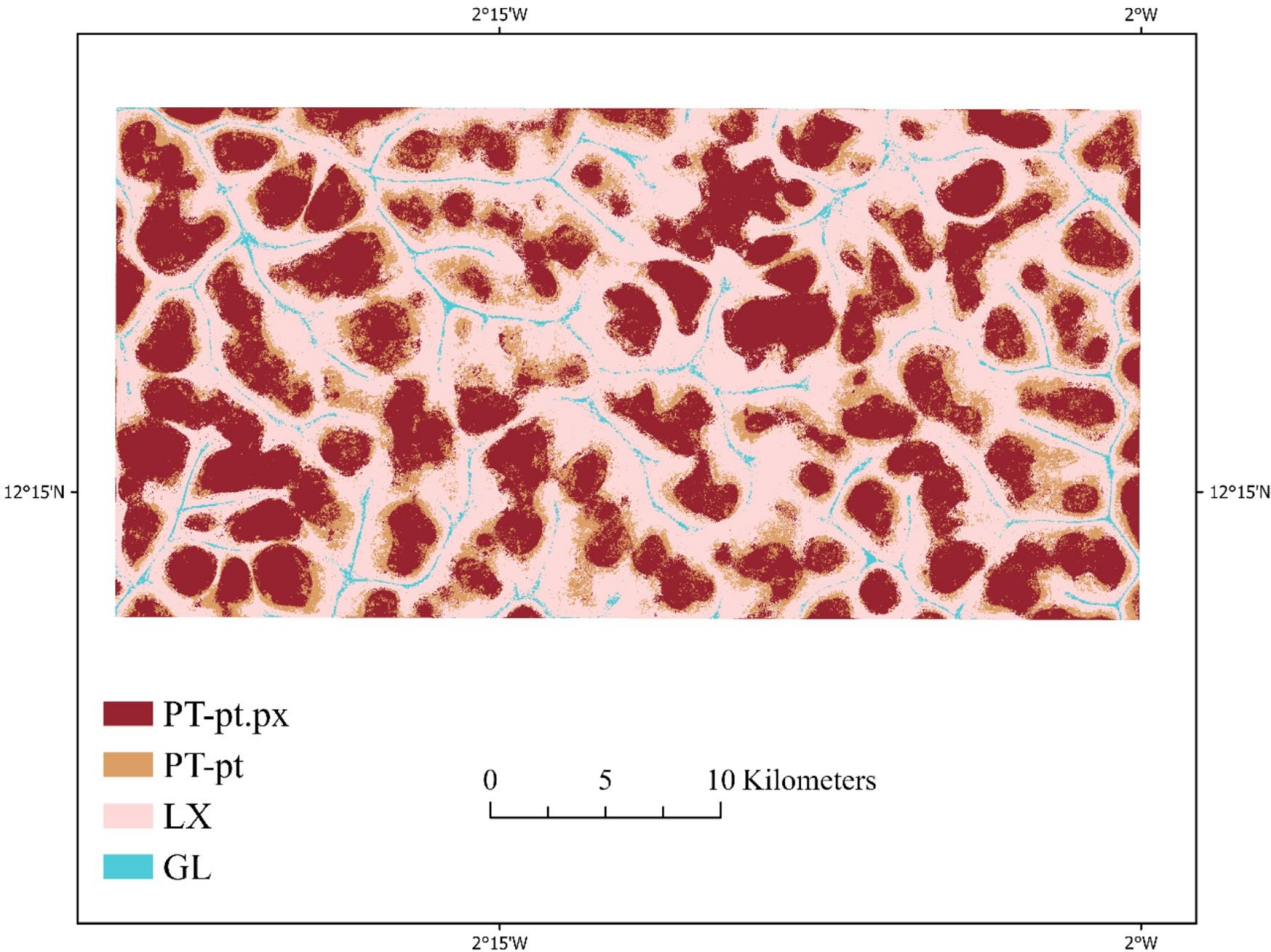
Soil map of the study area generated using the best-performing XGBoost model (Scenario 5), illustrating the spatial distribution of the four major soil types derived from multisource remote sensing and topographic information.

**Fig. 7:**
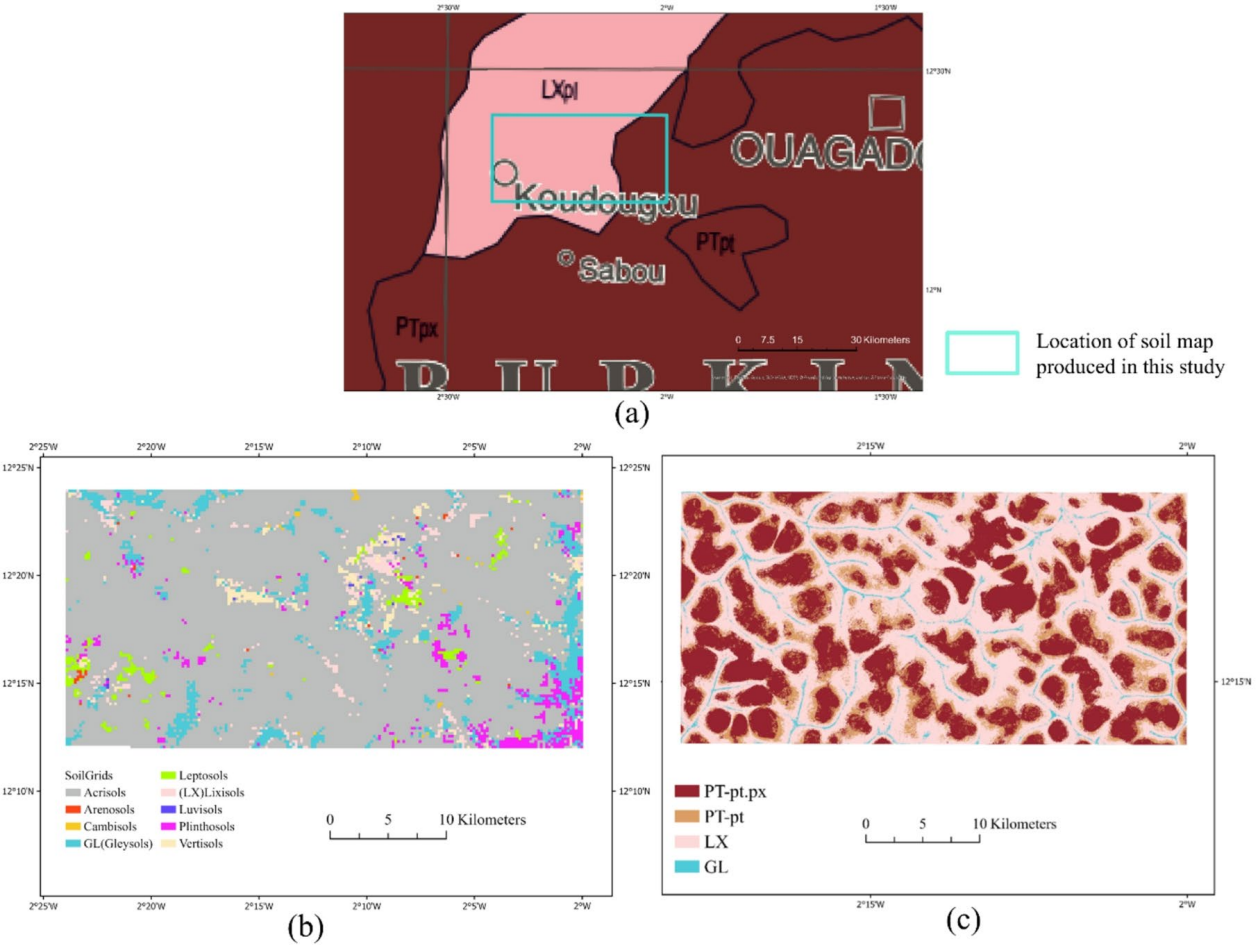
Highlighting improved soil distribution for the study area across three sources: (**a**) Soil Atlas of Africa soil map published in 2013 (original scale 1:3,000,000), (**b**) SoilGrids-250 m, and (**c**) Soil classification map generated in this study.

Using the best-performing XGBoost model and the selected set of feature combinations (Scenario 5), we produced a high-resolution soil type map of the study area (Fig. 6). The map delineates the distributions of the four major soil types identified in the ground surveys: LX, PT-pt, PT-pt.px, and GL. The spatial patterns of the soil types closely corresponded to the underlying terrain and spectral characteristics captured by Sentinel-2 and the topographic indices. For instance, LX was predominantly found near the river, where soil transported by rainwater is likely to be redeposited, consistent with higher TWI signatures. PT-pt was more common in moderately sloping areas, reflecting intermediate topographic positions, whereas PT-pt.px was distributed near or on the plateau. GL occurred in low-lying depressions with high moisture accumulation. Compared to the continental-scale Soil Atlas of Africa (2013) at 1:3,000,000 from European Soil Data Center and the global SoilGrids-250 m product (https://soilgrids.org/), our map provides a substantial improvement in spatial detail and classification accuracy (Fig. 7).

## Discussion

### Effectiveness of feature combinations and model selection in soil classification

Scenario 5 in Table 3provides complementary information that effectively captures the spatial variability of soil properties in this study. Feature importance analysis revealed that TWI was the most influential variable for soil classification in the study area. This dominance is consistent with the geomorphological and hydrological processes governing soil formation in this region. As described in Ikazaki et al^10^., soils in the Sudan Savanna landscape develop through slope-driven erosion on upper positions and the subsequent redeposition of materials downslope. Because the TWI represents the spatial pattern of water accumulation generated by rainfall, it effectively captures the erosion–deposition dynamics. Areas with a low TWI correspond to upper slopes where runoff and soil loss are prevalent, whereas areas with a high TWI reflect zones of water convergence where transported soil particles settle. These process gradients directly translate into differences in soil type, explaining why the TWI emerged as the strongest predictor. Winzeler et al^27^. demonstrated that TWI performance depends strongly on DEM quality, with DEM filtering or resampling substantially improving its correlation with soil moisture and thus soil water movement, in Hillslope Catena by examining field-collected soil volumetric water content. These findings support the importance of the TWI in this study, particularly in differentiating soil groups such as GL, Plinthosols, and LX.

Some studies have explored the application of Sentinel-1 and 2 products in soil property and soil moisture mapping and revealed their effectiveness using machine learning models^58–60^. Consistent with these findings, this study also showed that SWIR bands, particularly band 12, of Sentinel-2 were also highly informative for effective discrimination between soil types based on their spectral responses. In particular, the combination of terrain and spectral features provided complementary information that is critical for accurate soil classification in heterogeneous savanna landscapes. While TWI served as the primary discriminant factor reflecting topographic controls on soil formation, multispectral features derived from Sentinel-2 contributed mainly to refining the boundaries between soil classes. This suggests that terrain-driven hydrological processes form the fundamental framework controlling soil distribution in the Sudan Savanna, whereas spectral information improves the delineation of class transitions and local variability. In addition, it should be noted that the Sentinel-1 SAR-combined scenarios also produced relatively strong coefficients of determination values, but these were slightly lower than those of the scenarios with Sentinel-2 data. This is likely due to the relatively homogeneous surface roughness and sparse vegetation cover in the study area during the dry season, which reduced backscatter variability between soil types.

Regarding model selection and configuration, this study prioritized model robustness and generalization over extensive hyperparameter optimization, given the limited and imbalanced dataset. Default or commonly recommended parameter settings were adopted to reduce the risk of overfitting and to ensure fair comparison among algorithms. Among three ML models, the superior performance of XGBoost highlights its ability to capture complex nonlinear relationships and interactions between spectral and terrain features, and its built-in regularization prevents overfitting in small datasets. Ma et al^59^. also showed that XGBoost performed better in mapping soil salinization than the CART and RF models. In our study, RF performed reasonably well but was somewhat less effective in modeling subtle feature interactions compared with XGBoost. The SVM model showed moderate performance across the evaluated scenarios. With feature scaling applied to the predictor variables, the inclusion of topographic information improved SVM accuracy, particularly in scenarios incorporating TWI. However, its overall performance remained lower than that of the ensemble models. This may be attributed to the sensitivity of SVM to high-dimensional feature spaces and correlated predictors, which can complicate the identification of optimal decision boundaries in multiclass classification problems. Overall, these findings demonstrate that combining spectral and topographic information enhances soil-type discrimination and that ensemble learning models, especially XGBoost, are particularly well suited for capturing the heterogeneity of savanna landscapes. Future work may explore systematic hyperparameter optimization and automated search strategies to further enhance model performance.

### Contribution of remote sensing soil classification to agricultural management

The accurate soil maps produced in this study illustrate the potential of combining remote sensing and machine learning to support sustainable agricultural practices in the Sudan Savanna. Field-scale soil type information, which was previously unavailable, enabled local farmers and land managers to make informed decisions regarding soil and crop management practices based on the underlying soil characteristics. The Soil Atlas of Africa (Fig. 7a) depicts highly generalized mapping units covering broad soil regions, within which local variability cannot be resolved. Similarly, the SoilGrids-250 m map (Fig. 7b) represents the study area using multiple soil classes at a coarse grid resolution, resulting in fragmented and spatially smoothed patterns that are not well aligned with field-scale soil variation. Although the distribution of GL was broadly consistent between SoilGrids and the present study, large areas mapped as LX, PT-pt, and PT-pt.px in this study are predominantly misclassified as Acrisols in SoilGrids. In contrast, the map produced in this study resolved fine-scale transitions in the soil distribution, reflected locally verified classifications, and aligned more closely with the topographic and hydrological processes observed in the landscape. This enhanced spatial resolution enabled the identification of management-relevant soil boundaries that were previously indistinguishable, thereby offering more actionable information for farmers, extension services, and land-use planners.

Ikazaki et al. (2025) demonstrated that soil type is an important factor, along with effective soil depth, which has a crucial impact on the agronomic recommendations for sorghum in Africa. They emphasized the necessity of soil maps with higher resolution, which can provide local farmers with soil information. Iizumi et al^61^. conducted a crop choice analysis using a machine learning approach and highlighted that soil types influence the yields of various crops, such as cowpea, groundnut, soybean, maize, millet, and sorghum, in the Sudan Savanna. Therefore, the detailed soil map produced in this study can guide optimal crop placement and provide updated soil information for local livelihoods. Moreover, the integration of freely available Sentinel-1 and Sentinel-2 imagery with a terrain-derived index provides a cost-effective and scalable approach for soil mapping in data-scarce regions. This study demonstrates that machine learning models, particularly ensemble methods such as XGBoost, can effectively process multisource data to generate actionable information for agricultural planning and environmental management.

### Limitations and future work

Although this study produced promising results, it had some limitations that need to be considered for future improvement. First, the number of ground-truth soil locations (141 points) was relatively small, which may limit the generalizability of the models across the broader Sudan Savanna, although the soil-forming factors in this region were similar across large areas. Increasing the number of field samples would enhance model robustness and allow for finer discrimination among soil types. Another limitation of this study is the imbalance in the ground reference dataset, particularly the small number of Gleysols samples, which reflects their limited spatial representation in the study area. Such class imbalance can influence model training by biasing predictions toward majority classes and may lead to unstable or less reliable estimates of performance metrics for minority classes. This constraint is closely related to the validation strategy. Because the available samples are not only limited in number but also spatially clustered along transects, we employed LOOCV to maximize the use of field observations for training. However, when samples are spatially close, LOOCV may allow spatial autocorrelation leakage, meaning that training and validation samples share similar environmental conditions. This can result in more optimistic accuracy estimates compared with spatial cross-validation approaches, which explicitly separate data in geographic space^62–64^. However, the limited number of ground reference samples, especially for minority soil classes, means that spatial cross-validation would substantially reduce the effective training data per fold and could cause unstable model fitting or missing classes in training sets. In this context, accuracy estimates may be more constrained by sample scarcity than by model generalization. Future studies with larger and more spatially distributed datasets should adopt spatial cross-validation to obtain more spatially robust accuracy assessments. Future work should prioritize additional field sampling of underrepresented soil types to enhance model robustness and enable more reliable accuracy assessment.

In addition, this study relied on a commercially available 5 m AW3D DEM to derive the TWI. Although this method provides high-resolution terrain information, accessibility may be limited in some regions. Testing our proposed method with freely available global DEMs with 30 m spatial resolution, such as ALOS or ASTER, would make the approach more widely applicable and suitable for large-scale mapping efforts. Because the 30 m resolution is still much smaller than the size of the fields of local farmers, changing the resolution from 5 m to 30 m is unlikely to significantly affect the accuracy of soil classification.

Another limitation arises from the nature of satellite remote sensing itself. Optical and radar sensors primarily capture surface characteristics and have limited ability to detect detailed soil properties or subsurface layers. Consequently, information on deep soil profiles, textural variability at depth, and certain chemical properties cannot be obtained directly from these datasets. Integrating ground-based measurements or proximal sensing techniques could complement satellite-derived data and improve soil characterization. Future studies could expand the classification scheme, integrate temporal satellite data to account for seasonal variations, and explore hybrid modeling approaches that combine machine learning with process-based soil models to improve interpretability and agricultural relevance.

## Conclusion

This study focused on the Central Plateau of Burkina Faso, a representative area of the Sudan Savanna. Remote sensing predictors included Sentinel-1 radar, Sentinel-2 spectral bands, vegetation indices, and the TWI. Using these data, three machine learning models, RF, XGBoost, and SVM, were trained and evaluated to compare their effectiveness in soil classification. These findings contribute methodologically and practically to the literature. From a methodological perspective, this study assessed the performance of different machine learning algorithms when integrating multisource datasets in a complex savanna environment. Practically, it delivers updated soil-type information that can support agricultural planning, soil management, and environmental monitoring in the study area. More broadly, this study highlights the value of combining optical, radar, and topographic information for digital soil mapping in data-limited regions. Given the limited spatial extent and transect-based sampling design of this study, further validation across larger and more diverse landscapes is recommended to fully assess the transferability of the proposed framework to other parts of the Sudan Savanna and similar environments.

## Data Availability

The datasets generated and analyzed in the current study are available from the corresponding authors upon reasonable request for academic and non-commercial research purposes, subject to project approval and data-sharing agreements.
